# Myco-Synthesis of Silver Nanoparticles and Their Bioactive Role against Pathogenic Microbes

**DOI:** 10.3390/biology12050661

**Published:** 2023-04-27

**Authors:** Ahmed Abdel-Hadi, Danish Iqbal, Raed Alharbi, Sadaf Jahan, Omar Darwish, Bader Alshehri, Saeed Banawas, Manikanadan Palanisamy, Ahmed Ismail, Sahar Aldosari, Mohammed Alsaweed, Yahya Madkhali, Mehnaz Kamal, Faria Fatima

**Affiliations:** 1 Department of Medical Laboratory Sciences, College of Applied Medical Sciences, Majmaah University, Majmaah 11952, Saudi Arabia; a.abdelhadi@mu.edu.sa (A.A.-H.); s.jahan@mu.edu.sa (S.J.); b.alshehri@mu.edu.sa (B.A.); s.banawas@mu.edu.sa (S.B.); m.palanisamy@mu.edu.sa (M.P.); s.aldosari@mu.edu.sa (S.A.); m.alsaweed@mu.edu.sa (M.A.); y.madkhali@mu.edu.sa (Y.M.); 2 Botany and Microbiology Department, Faculty of Science, Al-Azhar University, Assiut Branch, Assiut 71524, Egypt; 3 Department of Health Information Management, College of Applied Medical Sciences, Buraydah Private Colleges, Buraydah 51418, Saudi Arabia; 4 Department of Public Health, College of Applied Medical Sciences, Majmaah University, Majmaah 11952, Saudi Arabia; r.abdullah@mu.edu.sa (R.A.); ai.ismail@mu.edu.sa (A.I.); 5 Department of Mathematics and Computer Science, Texas Women’s University, Denton, TX 76204, USA; odarwish@twu.edu; 6 Department of Biotechnology, Faculty of Agriculture, Al-Azhar University, Cairo 11751, Egypt; 7 Department of Pharmaceutical Chemistry, College of Pharmacy, Prince Sattam Bin Abdulaziz University, Al-Kharj 11942, Saudi Arabia; mailtomehnaz@gmail.com; 8 Department of Agriculture, Integral Institute of Agricultural Science and Technology, Integral University, Lucknow 226026, India

**Keywords:** biomedical, drug resistance, fungal extract, microbicidal, nanotechnology, silver nanoparticle

## Abstract

**Simple Summary:**

The relatively high prevalence of microbial infections and the rising resistance to traditional antibiotics are the causes of the need for revolutionary antibiotics. Nanotechnology, a technique that employs materials featuring nanometer size, has grown in popularity for therapeutic uses and is very intriguing as a means of eradicating or limiting the activity of several pathogens. Silver nanoparticles have been shown to have antimicrobial properties against fungi and bacteria. The unique properties of silver nanoparticles, such as their high surface-area-to-volume ratio and the ability to release silver ions, can cause damage to the microbial cell membrane, interfere with cellular processes, and make them effective against a wide range of microorganisms. Synthesis of nanoparticles via natural products could potentiate their therapeutic activities. Moreover, phosphatase enzyme is also known to possess antimicrobial effects, and there is a fungus (*Fusarium oxysporum*) reported to have phosphatase enzymes in its extracellular fluid. Therefore, we focused on synthesizing silver nanoparticles by using extracellular proteins released by *Fusarium oxysporum* and thereafter evaluated its biological activities against pathogenic microbes. Our findings illustrated that synthesized nanoparticles showed prominent anti-microbicidal activities against various pathogenic bacterial and fungal species. Thus, these nanoparticles may be used against drug-resistant infections.

**Abstract:**

Nanotechnology based on nanoscale materials is rapidly being used in clinical settings, particularly as a new approach for infectious illnesses. Recently, many physical/chemical approaches utilized to produce nanoparticles are expensive and highly unsafe to biological species and ecosystems. This study demonstrated an environmentally friendly mode of producing nanoparticles (NPs) where *Fusarium oxysporum* has been employed for generation of silver nanoparticles (AgNPs), which were further tested for their antimicrobial potentials against a variety of pathogenic microorganisms. The characterization of NPs was completed by UV–Vis spectroscopy, DLS and TEM, where it has been found that the NPs were mostly globular, with the size range of 50 to 100 nm. The myco-synthesized AgNPs showed prominent antibacterial potency observed as zone of inhibition of 2.6 mm, 1.8 mm, 1.5 mm, and 1.8 mm against *Vibrio cholerae, Streptococcus pneumoniae, Klebsiella pneumoniae* and *Bacillus anthracis,* respectively, at 100 µM. Similarly, at 200 µM for *A. alternata, A. flavus and Trichoderma* have shown zone of inhibition as 2.6 mm, 2.4 mm, and 2.1 mm, respectively. Moreover, SEM analysis of *A. alternata* confirmed the hyphal damage where the layers of membranes were torn off, and further EDX data analysis showed the presence of silver NPs, which might be responsible for hyphal damage. The potency of NPs may be related with the capping of fungal proteins that are produced extracellularly. Thus, these AgNPs may be used against pathogenic microbes and play a beneficial role against multi-drug resistance.

## 1. Introduction

The problems faced by healthcare systems nowadays are mostly related to multi-drug resistance (MDR), which poses a serious threat to public health. Microbial responses towards standard medication fail because of the rapid formation of unique mechanisms of resistance and a loss in the effectiveness of treating serious infections, which leads to prolonged illness, higher medical expenses, and a higher mortality risk [1,2]. Bacterial resistance is brought about by changes in how microorganisms react to antibacterial medicines, either by rendering them inactive or by reducing their therapeutic efficiency. Due to genetic changes throughout time, these resistances develop spontaneously in microbes. Such alterations are greatly favored by the improper and abusive use of antibiotics. This causes infection periods to last longer, death rates to rise and the financial load on health systems to grow. There are several different ways that microbes might become resistant to antibiotics, including enzymatic methods (lactamases, acetyltransferases or aminoglycoside modifying enzymes). Another prevalent resistance mechanism, along with antibacterial modifications, targets altering membrane permeability, which prevents the entrance of the antimicrobial drug (mutations within DNA gyrase/topoisomerase enzymes) [3]. Thus, MDR developed and spread more frequently because of incorrect antimicrobial medicine use, unhygienic environments, poor food handling, antibiotics abuse and inefficient healthcare-associated infections strategies [4]. To combat microbial resistance, a variety of measures have been used, including the application of bactericidal agents, emerging novel antibiotics and combined treatment [5,6,7]. Nanotechnology is the science, engineering and application of materials and devices with dimensions on the nanoscale, typically ranging from 1 to 100 nanometers (nm). At this scale, the properties of materials can differ significantly from those at larger scales, allowing for the creation of new materials and devices with unique properties and functions. Nanotechnology has the potential to revolutionize many areas of science and technology, including electronics, energy, medicine, and materials science. Researchers are exploring the use of nanotechnology to create more efficient and powerful computer processors, sensors, and batteries, as well as new drug delivery systems and diagnostic tools for medicine [8]. Nanoparticles have shown great promise in the fight against bacterial infections. Some nanoparticles have inherent antibacterial properties, while others can be designed to target specific bacteria or even disrupt bacterial biofilms. Silver nanoparticles, for example, have been shown to be effective against a wide range of bacteria, including drug-resistant strains. These nanoparticles work by disrupting the bacterial cell membrane and interfering with cellular processes [9]. Other nanoparticles, such as zinc oxide nanoparticles, have been shown to disrupt biofilms and reduce bacterial growth [10].

Multiple attributes of NPs make them advantageous as carriers of medications to fight disease-causing microorganisms. These qualities include improved drug absorption and stability, ease in synthesis, interaction with target chemicals and regulated release that can be influenced by stimuli such as temperature, light and pH. Nanoparticles have incredibly small size and high surface-to-volume ratio that enable their special functioning in drug delivery. Use of NPs in the management of infections brought on by pathogenic microbes and MDR variants represents a significant competitive advantage over traditional medicines. Silver nanoparticles (AgNPs) are thought to be the most efficient nanoparticle against pathogenic microbes. Many antibiotics have poor membrane transport; these drug-loaded NPs vehicles can penetrate within host cells via endocytosis, making their intracellular penetration easier. Moreover, there is various evidence that exposing microbes to these NPs may improve their tolerance towards antibiotics [11,12]. Hence, silver nanoparticles are one of the promising approaches for the management of pathogenic microbes by preventing bacterial cells’ efflux pumps, restoring the bactericidal activity of traditional antibiotics, and decreasing the ability of microorganisms to produce biofilms [6,13,14]. These are of great importance to cure the various pathophysiological disorders due to their unique biological, physical, and chemical properties [15,16,17].

Two alternative methods, namely the top-down technique and the bottom-up approach, can be used to synthesize NPs. Additionally, three various approaches, including physical, chemical, and biological procedures, are used to create NPs. The top-down strategy is used by the physical methods, whereas the bottom-up approach is used by the chemical as well as biological approaches to synthesize NPs. Some of the most popular physical processes used to create NPs are laser ablation, evaporation–condensation, electrolysis, diffusion, sputter deposition, laser ablation, pyrolysis, plasma arcing and high-energy ball milling. The main drawbacks of these procedures are their poor output rates, costly operations, and significant energy usage [18]. The traditional and most popular methods for the formation of metallic NPs are to use chemical synthesis methods, such as micro-emulsion/colloidal, chemical reduction, electrochemical and thermal degradation, where chemical reduction of NPs from their respective metal salt precursors is completed by adding specific reducing agents. Various stabilizing agents (dodecyl benzyl sulphate and polyvinyl pyrrolidone (PVP)) and reducing agents (formaldehyde, sodium borohydride, methoxy polyethylene glycol, potassium bitartrate and hydrazine) can be used, but their usage results in the formation of undesirable byproducts [19]. As a result, there is a growing need for approaches for metallic NPs that are dependable, eco-friendly, high-yielding, and sustainable. Natural products and their derivatives were found to have better therapeutic potentials against several metabolic and infectious disorders, such as diabetes, oxidative stress, hyperlipidemia, ulcers, neurodegenerative disorders, cancer, and microbial infections [11,15,17,20,21,22,23,24,25,26,27,28,29,30,31,32,33,34,35]. Numerous therapeutic effects of natural products and metallic nanoparticles were previously reported to manage the deleterious effects of pathophysiology related to oxidative stress, microbial infection, and inflammation [13,20,36,37]. The green synthesis process provides an economical, reproducible, fast, and ecologically sustainable way to create metallic nanoparticles. Myco-nanotechnology is an emerging scientific field that studies how fungi might produce nanomaterials or nanostructures with suitable forms and sizes. In comparison to bacteria, fungi have a stronger tolerance to the flow pressure and agitation of the bioprocesses, making them a better choice for industrial-scale synthesis of metal-based nanomaterials. These applications could help provide incremental solutions through green chemical methods. Silver nanoparticles (AgNPs) have special optical, catalytic, and antimicrobial properties that are attracting significant research and commercial interest [38,39,40]. In this post-antibiotic era, AgNPs have been investigated to find novel therapeutics that can help to combat harmful microbes without fostering the emergence of new resistances. AgNPs can be considered as a great alternative as they may be used to fight infection. Infections caused by antibiotic-resistant microbes are an issue for the entire world. This application for an alternative to antibiotics has received much study recently, with the goal of creating novel antimicrobials for detoxification or infection treatments while utilizing the knowledge previously known about their effectiveness even against MDRs. However, the elimination of bacteria may be attributed to the silver nanoparticles’ ability to continuously discharge silver ions. Silver ions can cling to the cell wall and cytoplasmic membrane due to electrostatic attraction and affinity to sulphur proteins. The bacterial envelope may be damaged because of the adhering ions increasing the cytoplasmic membrane’s permeability. Respiratory enzymes may be inactivated following the import of free silver ions into cells, leading to the formation of reactive oxygen species but not adenosine triphosphate [41]. Cell membrane breakdown and DNA alteration can both be triggered by reactive oxygen species as a primary agent. Additionally, by denaturing ribosomes in the cytoplasm, silver ions can prevent the production of proteins.

Numerous studies have focused on the biological creation of metal nanoparticles; however, the ability of the *Fusarium oxysporum* with good phosphate-solubilizing potential [42] to be utilized to prepare the silver nanoparticles remains insufficiently explored [43]. Thus, the main purpose of this research is to employ the novel strain of fungus *Fusarium oxysporum* extracellularly to execute silver nanoparticle production as well as to examine the efficacy of these myco-synthesized NPs for anti-microbicidal action against pathogenic organisms.

## 2. Materials and Methods

### 2.1. Synthesis of AgNPs from F. oxysporum

The fungus was grown on MGYP broth [Hi-media], which included malt extract, glucose, yeast extract and peptone [0.5%, 1%, 0.3% and 0.5%] maintained at 28 °C for 72 h before being collected by filtering through a polypropylene sieve. After that, 15 g of mycelial threads were obtained using Whatman filter paper and washed with sterilized water, which then transferred to 150 mL of deionized water. The biomass was further allowed to agitate at 150 rpm for 72 h at 28 °C and pH 7.2 in an Erlenmeyer flask for secretion of extracellular protein [13]. The procedure was allowed to proceed in the Erlenmeyer flasks with the formation of silver nanoparticles with the addition of 1 mM silver nitrate [AgNO_3_]. Using an ultraviolet–visible spectrophotometer, the time-dependent production of AgNPs was investigated (Beckman DU-20 spectrophotometer). A negative control of 1 mM silver nitrate was employed, whereas a positive control of salt-free supernatant was used.

### 2.2. Characterization of Nanoparticles

#### 2.2.1. Ultraviolet–Visible Spectroscopy (UV–Vis Spectroscopy)

Beckman DU-20 spectrophotometer was used to monitor the emergence of reduced silver nanoparticles in colloidal suspension. For AgNPs, the absorbance spectrum of the NPs sample was measured in the 300–600 nm range [13,44]. The results have been examined and recorded further using the “UVWinlab” application. As a benchmark, distilled water was employed with a Beckman DU-20 UV–Vis spectrometer. Salt-free supernatant was used as a negative control.

#### 2.2.2. Differential Light Scattering (DLS)

Monitoring dynamic variations in light scattering intensity brought on by the Brownian particle movement, the sample was equilibrated at 25 °C for 5 min before being measured with a Malvern-Zetasizer-Nano-ZSTM (Malvern Instruments Ltd., Malvern, UK) and Dispersion Technology Software v.5.1 (Malvern Instruments Ltd.). The measurement provided the diameter distribution’s peaks, the average diameter, and the polydispersity index (PdI), which characterized the amplitude of the variation in particle size. The PdI scale has a range of 0 to 1. With a temperature equilibration time of 1 min at 25 °C, each measurement was carried out twice. An ultra-sonic bath (ULTRA sonic, Capistrano Beach, CA, USA at 57 X, 50/60 Hz) was used to extract AgNPs in deionized water (dH_2_O). At 24 °C, dH_2_O has a viscosity of 0.877 cP (A&D Instruments Ltd., Oxfordshire, UK). AgNPs dH_2_O had a 1.330 refractive index [5,13].

#### 2.2.3. Transmission Electron Microscopy (TEM) Analysis

Produced AgNPs were characterized using TEM, which involved placing the particles on a gold-coated negative grid and evaporating the solvent. The TEM study was carried out using a Perkin-Elmer model [JEM-1000; JEOL (UK) Ltd., Welwyn Garden City, UK], which was powered at a 1000 kV accelerating voltage [5].

#### 2.2.4. Fourier-Transform Infrared Spectroscopy (FTIR)

FTIR findings from infrared spectroscopy of bio-transformed compounds identified in supernatant were refrigerated and preserved in potassium bromide at a ratio of 1:100. The diffuse reflectance mode at (DRS-800) linked with FTIR instrument was used to record the FTIR sample spectra (Digital Excalibur 3000 series, Excalibur, Tokyo, Japan). In the range of 400–4000 cm^−1^, all parameters were measured at a resolution of 4 cm^−1^ [45].

### 2.3. Antibacterial Activity

*Streptococcus pneumoniae* [NCMR, accession number MCC 2425], *Klebsiella pneumoniae* [NCIM, accession number 5432], *Vibrio cholerae* [ATCC 15748], *Bacillus anthracis* [NCMR, accession number MCC4453] used in this study were procured from National Centre for Cell Science (NCCS). Initially the strains were kept at 4 °C in nutrient agar slants and thereafter, at the time of study, the subcultures were further transferred to NB media [Hi-media] for antibacterial assessment of NPs, respectively [5]. The turbidity of bacterial inoculums used were adjusted at 0.8, equal to ~1 × 10^8^ CFU/mL analyzed at OD_600_. The agar well-diffusion technique was used to examine biologically synthesized AgNPs at different concentration (15–100 μM), antibiotic [doxycycline] at concentration 50 μg/mL [Hi-media], AgNPs + Antibiotic [doxycycline] at concentration 50 μM + 50 μg/mL, for antimicrobial properties. Each assay was performed three times, and, in each case, the average zone of inhibition, eliminating well, was noted. As a negative control for measurements of AgNPs, salt-free supernatant was used.

### 2.4. Antifungal Activity

Agar well-diffusion technique was used to assess antifungal efficacy of AgNPs towards pathogenic fungus *A. flavus* [NCIM, accession number 1316], *A. alternata* [NCIM, Accession No:718] and *Trichoderma sp* [NCIM, Accession No:1458] procured from National Center of Cell Science [NCCS]. Fungal suspensions were prepared in sterilized phosphate buffer solution (PBS) pH 7.0, and then the inoculums were adjusted to 10^7^ spores/mL counted in a cell counter chamber. One mL of each fungal suspension was uniformly distributed onto PDA Plates. Sterile Cork-borer was used for making wells (5 mm), and the wells were aseptically filled with AgNPs at several concentration (75–200 μM), Antibiotic [Amphotericin] at 50 μg/mL [Hi-media], AgNPs+Antibiotic [Amphotericin] at 75 μM + 50 μg/mL and the plates were then incubated for 7 days at 28 ± 4 °C. The average inhibition zone was estimated for each case [5]. As a negative control for measurements of AgNPs, salt-free supernatant was used.

### 2.5. Preparation of Cells for SEM Analysis

Cell morphology was investigated using scanning electron microscopy (FESEM MODEL NO. GEMINISEM 300) to evaluate the cellular changes induced by AgNPs in *A. alternata*. The cultures were subjected to laser light for 5 min to investigate the morphological alterations in fungal mycelia caused by silver nanoparticles (Ag-NPs) at 100µM. Untreated samples act as a control and were maintained in nutritional medium. After being washed, the microbial cells were resuspended in PBS. Samples were placed upon membrane filters and fixed for 4 h in 2% (*v*/*v*) glutaraldehyde before being washed twice in PBS and fixed for 1 h in 1% (*w*/*v*) osmium tetra oxide. The solvent was removed with an analytical graded ethanol series at different percentage, i.e., 30%, 50%, 70%, 80%, 90% and 100%, dried using liquid CO_2_ and then plated with a gold coater. A SEM apparatus linked to an energy-dispersive X-ray microscope-EDX (OCTANE ELECT PLUS) was used to determine the semi-quantitative chemical composition of the samples [46].

### 2.6. Statistical Analysis

The findings of each test were run in triplicate, and the mean and standard deviation were used to express them. One-way variance analysis (ANOVA) with Dunnett’s test was employed for repeated comparison testing of normally distributed samples with homogeneous variance. The threshold for statistical significance was set at *p* < 0.05.

## 3. Results and Discussion

### 3.1. Synthesis of Fungal-Mediated Nanoparticles

**Figure 1:**
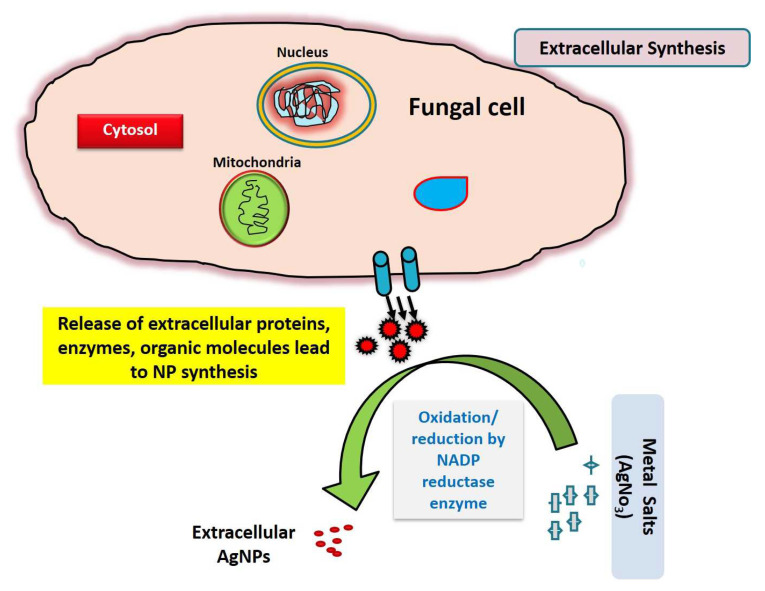
Schematic representation of extracellular mode of reduction of metallic salt/ions by fungal extracellular secretion.

Due to the enormous ease of handling, proteins generated, yields and release of minimal cytotoxicity of the residues, fungal sources are found to be promising reducing sources in the production of nanoparticles [43]. Since they are simple to deal with in the downstream process, these microorganisms have been advocated as a safer choice for large-scale green nanoproduction. It has been demonstrated that various proteins and other biomolecules released by microorganisms cause capping and stability of nanoparticles, which may be employed in drug synthesis, biofertilization and in microbial degradation [47]. Due to capping proteins, the stability of green-synthesized AgNPs may have an added benefit as antimicrobial agents. The silver nitrate was bio-reduced by the fungus *F. oxysporum*, resulting in silver nanoparticles visible by visual observation within the reaction flasks (Figure 1).

**Figure 2:**
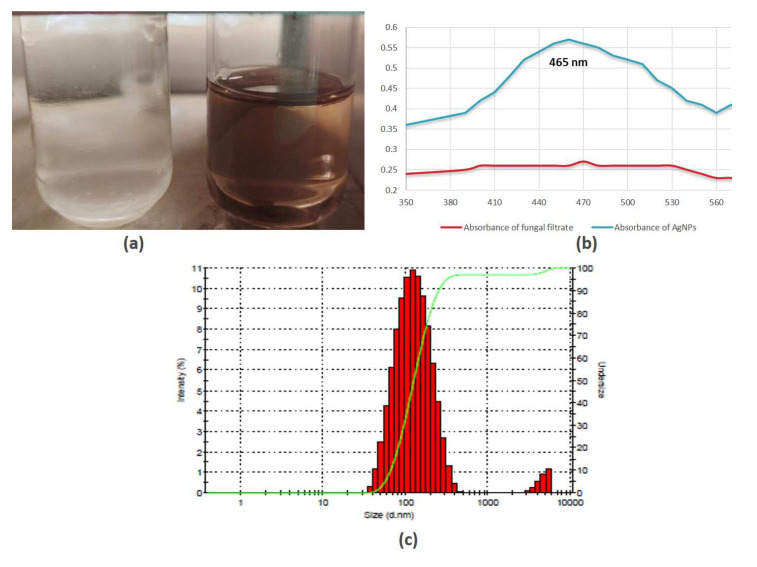
Silver nanoparticles formation based on (**a**) color change due to surface plasmon resonance; (**b**) UV–Vis spectroscopy; (**c**) dynamic light scattering (DLS).

The aqueous silver nitrate solution remained colorless prior to the reaction, as illustrated in Figure 2a, and these silver ions were bio-reduced into a characteristic brown color after incubation with the fungal cell-free solution. This color shift was caused by the extracellular creation of nanoparticles, which includes trapping metallic ions on the cell membrane and decreasing ions that might be due to the presence of reducing enzymes [48].

### 3.2. Spectrophotometric and Differential Light Scattering Analyses of Silver Nanoparticles

Spectroscopy study reveals no indication of absorbance for fungal extract in the 300–600 nm spectrum; however, fungal extracts subjected to salts (AgNO_3_) exhibited significant absorption, with a peak at 460 nm, which indicates the formation of AgNPs (Figure 2b). Similar findings were observed in previous studies where it has been shown that the absorbance of silver NPs was nearly at 420–460 nm, respectively [40,49]. The size distribution of NPs analyzed through DLS spectra demonstrated that the size of AgNPs is in the range between 30 nm and 300 nm (Figure 2c), and the highest % intensity was observed for particle size between 80 and 110 nm. The synthesized AgNPs are polydisperse in form as the PDI values are 0.3. A previous study showed that biologically mediated silver nanoparticles represent similar findings of DLS data and particle size distribution [50].

In the presence of protein, metallic salt oxidation turns into their respective nanostructures, which is mainly responsible for the size variation. The particle size may be determined using this approach by estimating random fluctuations in the intensity of light diffused from colloidal suspension.

### 3.3. FTIR and TEM Analyses

**Figure 3:**
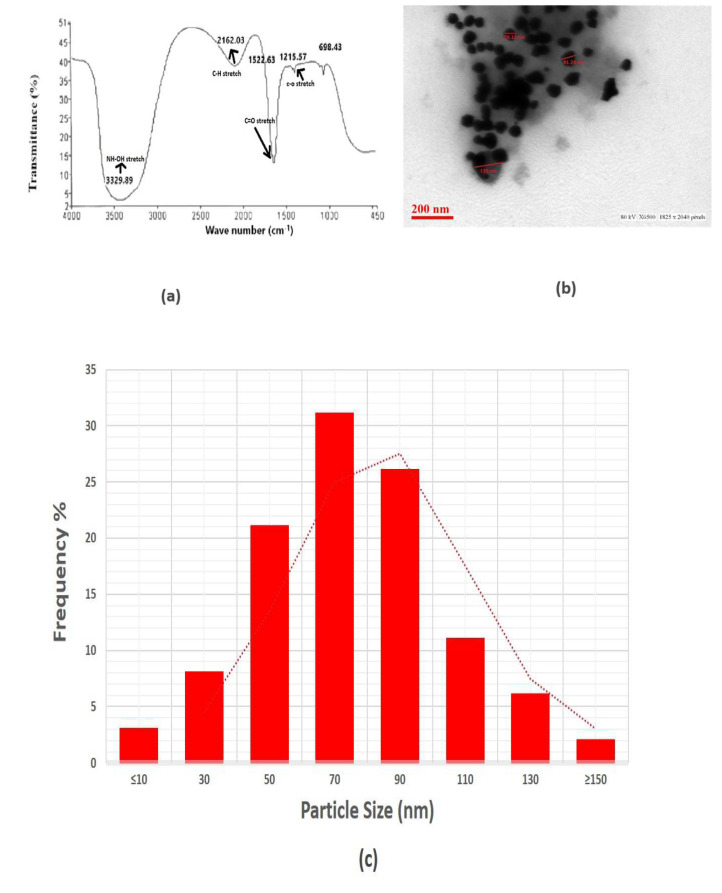
(**a**) FTIR analysis. (**b**) Transmission electron microscopic analysis. (**c**) Particle size distribution.

The silver nanoparticles synthesized by using *F. oxysporum* were subjected to FTIR analysis to find out the bioactive compounds synthesized by the fungus and associated with the nanoparticles. Several bands were observed in the region 400–3700 cm^−1^ centered at 1215.57, 1522.63, 2162.03 and 3329.89 cm^−1^ peaks observed in the case of silver nanoparticles (Figure 3a).

The strong, sharp band centered at 1522.63 cm^−1^ is caused by amide group with carbonyl stretch (C=O) vibrations coupling of proteins released by the fungus. Research demonstrates that proteins can attach to nanoparticles via free amino groups or cysteine residues in the proteins, which then provide the nanoparticles stability by enclosing their surface. A weak broad band at 3329.89 cm^−1^ may represent the characteristic free hydroxyl group (-OH) of any quinone compounds secreted by this fungus. Isolation occurred of various anthraquinones that showed an absorption band at 3329.89 cm^−1^, where they could act as an electron shuttle to reduce the metal ions. The finding demonstrated that protein molecules can function as a reducing as well as stabilizing agent by interacting with AgNPs in the presence of free amino acids, cysteine sites or by electrostatic interaction of negative charges of carboxyl groups within the extracellular enzyme [51]. This demonstrates that the fungus utilized in this research may create molecules that resemble quinones, in which case it might function as an electron shuttle to reduce the metallic ions. The information related to FTIR analysis of fungal extracellular material for *F. oxysporum* has been reported earlier and showed similar peaks to those observed in our results [51,52]. These observations illustrated that the nanoparticles prepared in our study have capping of fungal extracellular matrix containing metabolites and proteins.

The representative TEM imaging (Figure 3b) clearly reveals the size and structure of the resulting nanoparticles, indicating that they are mostly spherical in shape. The revealed size of AgNPs is mostly from 50 to 100 nm, with an average diameter of 56 nm, but very few particles were also found to be less than 50 nm and higher than 150 nm, which supports our findings of DLS analysis, where we noticed the % intensity of some particles size distribution of around 30–50 nm and some of 100–300 nm. The particle size analysis was completed by calculating the diameter of the number of individual particles. The mean particle size was determined after fitting the histogram with a normal size distribution. The material synthesized by this green mode was found to be more homogeneous and have several particles of varying sizes. This result clearly shows the mostly spherical nature and different size range of particles (Figure 3c).

### 3.4. Antibacterial Property

**Figure 4:**
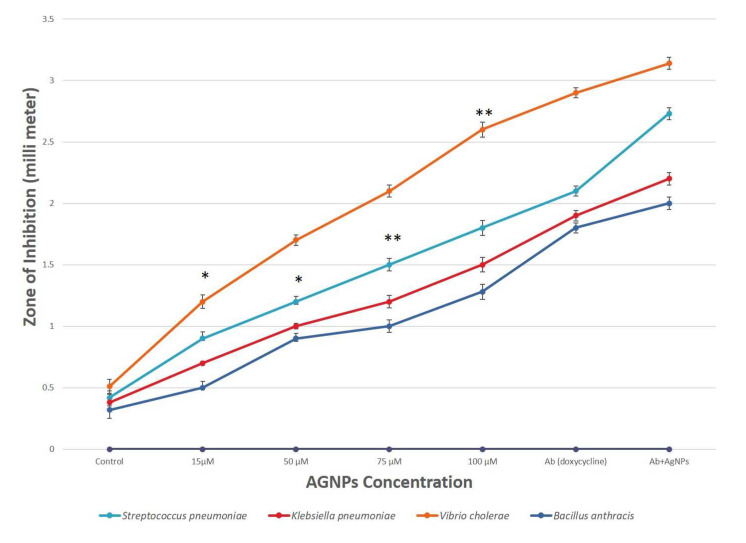
Comparative analysis of antibacterial activity of AgNPs for different pathogenic bacteria. Results are presented in relative units compared with controls. Data represents the mean ± standard deviation of three individual experiments. *, ** represent significantly different from control group (* *p* < 0.05; ** *p* < 0.01). Note: The *X*-axis shown as AgNPs concentration does not only have AgNPs but also shows control (fungal extract), antibiotic (Ab) and combination (Ab+AgNPs).

The agar well plate diffusion technique was used to examine the antibacterial properties of AgNPs against numerous harmful microorganisms, such as *S. pneumoniae, K. pneumoniae, V. cholerae* and *B. anthracis.* The inhibition zones of AgNPs at 100 μM against *Vibrio cholerae* and *Streptococcus pneumoniae* were found to be 2.6 and 1.8 mm, respectively. The pathogenic bacteria also showed a zone of inhibition when exposed to pure fungal extract, which was used as a control. The differences between the inhibitory zones observed by synthesized AgNPs and fungal extract have been shown in Figure 4; therefore, the values of zone of inhibition for fungal extracellular extract were considered as control and mentioned at nearly zero in the *Y*-axis of the graph. Thus, it can be interpreted that the AgNPs synthesized are responsible for the good antibacterial activity. When the concentration of AgNPs increased, a significant rise in antibacterial activity was demonstrated. Moreover, it was shown that using AgNPs in combination with the antibiotic (doxycycline) rather than by themselves increased their effectiveness, which is supported by a previous study [53]. The comparative analysis found that AgNPs had the highest antimicrobial activities against *V. cholerae*, followed by *S. pneumonia* and *K. pneumoniae* (Figure 4).

The mode of action of AgNPs against bacteria is not completely understood yet. However, several hypotheses are explaining the antibacterial activity of silver nanoparticles: (1) generation of reactive oxygen species; (2) release of Ag^+^ ions from AgNPs denaturize proteins by bonding with sulfhydryl groups; (3) attachment of AgNPs on bacteria and subsequent damage to bacteria. Recently, it has been shown that these alkaline phosphatase enzymes have antimicrobial efficacy against pathogenic microbes. A good amount of phosphate solubilization enzyme alkaline phosphatase is present in *F. oxysporum*; the nanoparticles created from them may exhibit gradual release of the encapsulated enzyme, which may possess antimicrobial efficacy [54,55].

### 3.5. Antifungal Activity

**Figure 5:**
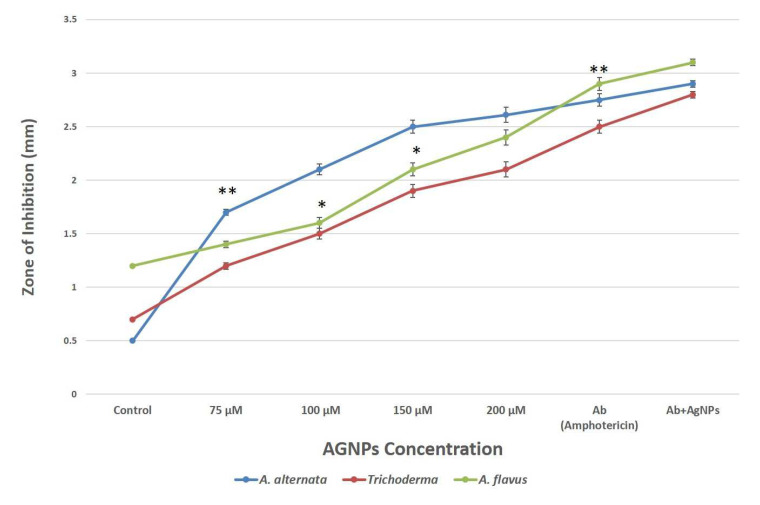
Comparative analysis of antifungal activity of AgNPs against pathogenic fungi. Data represents the mean ± standard deviation of three individual experiments. *, **, represent significantly different from control group (* *p* < 0.05; ** *p* < 0.01) Note: The *X*-axis shown as AgNPs concentration does not only have AgNPs but also shows control (fungal extract), antibiotic (Ab) and combination (Ab+AgNPs).

The results showed that AgNPs have substantial antifungal as well as inhibitory effects on colony development under in vitro conditions as they were discovered to suppress the development of fungus (*A. flavus, A. alternata* and *Trichoderma*) and produced an inhibition zone. The differences between the inhibitory zones observed by synthesized AgNPs and fungal extract are shown in Figure 5; therefore, the value of zone of inhibition for fungal extracellular extract was considered as a control and mentioned at nearly zero in the *Y*-axis of the graph. It was observed that the colony’s ability to develop decreases as AgNPs concentration rises. At a concentration of 200 μM, *A. alternata* showed an inhibition zone with a diameter of 2.6 mm, followed by *A. flavus*, which showed an inhibition zone of 2.4 mm, and *Trichoderma* with a diameter of 2.1 mm (Figure 5).

### 3.6. Scanning Electron Microscopy (SEM) and Energy-Dispersive Spectra (EDX)

**Figure 6:**
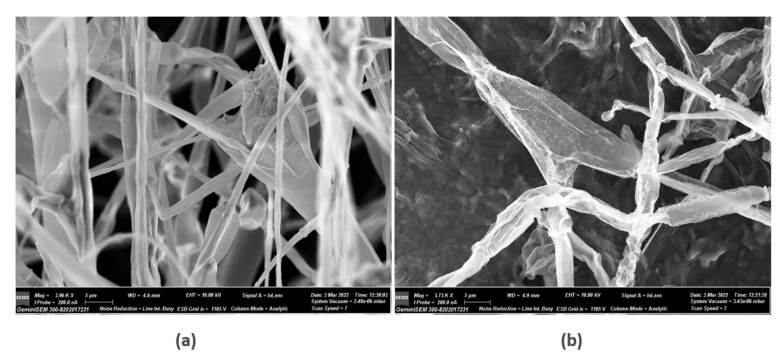
Scanning electron micrographs of *Alternaria alternata* mycelia after 5 days of cultivation at 26 °C: (**a**) control; (**b**) treatment with 150µM AgNPs.

Scanning electron microscopy was used to examine the inhibitory impact of AgNPs on the fungal isolates of *Alternaria alternata* that was cultured on PDA plates. AgNPs significantly destroyed the hyphae according to microscopic examination (Figure 6). When compared to the control, which displayed a consistent and smooth appearance, the SEM micrographs of *Alternaria alternata* mycelium before and after the treatment with AgNPs revealed compact AgNPs around the mycelium hyphal cell wall and significant morphological modifications. On the surface of the treated fungal hyphae, pores and pits could be observed. The interaction of AgNPs with substances that include phosphorus and sulphur is indeed a possible cause. Moreover, it has been thought that AgNPs can attach with negatively charged fungal membranes, rupturing cell walls and damaging the lipid bilayer of the membrane, causing intracellular ion efflux and death of cells [56,57].

**Figure 7:**
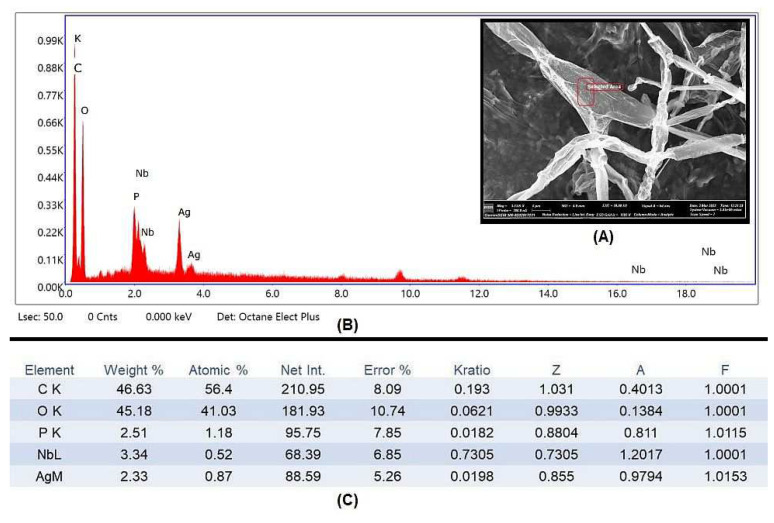
(**A**) ESEM micrographs of pathogenic *A. alternata* hyphae after treatment with AgNPs showing pores and cavities were formed on the surface. (**B**) The EDX spectrum for the nanoparticles visualized on the surface of *A. alternata* mycelium. (**C**) Elemental analysis of the EDX spectrum of synthesized AgNPs.

The element constitution of the synthesized AgNPs was examined using EDX (Figure 7A). Silver peaks were visible in the EDX spectra at energies of 3 and 3.7 keV, respectively. Previous reports also observed similar peaks, which could possibly be due to discharge of electron from the L and K shells of silver, respectively [58,59]. Hence, it is obvious from the EDX pattern that AgNPs are crystalline in form (Figure 7B). The growth media are high in carbon content, as shown in elemental analysis, which is a necessary factor for the fungus to perform its metabolic activity, while the other peaks may have been caused by inorganic impurities in the biomolecules from the fungal extract of *F. oxysporum* (Figure 7C).

## 4. Conclusions

This study demonstrated that the phosphate-solubilizing *Fusarium oxysporum* extract was extremely effective at reducing silver ions into silver nanoparticles. AgNPs produced by fungus were mostly spherical, having a size range of 50 to 100 nm, and were also used as effective biocontrol agents to reduce a variety of pathogenic bacterial and fungal strains. Maximum inhibition zones of 2.9 mm against *Vibrio cholerae* at 100 µM and 2.6 mm against *A. alternata* at 200 µM of AgNPs were observed, respectively; this shows that the biosynthesized NPs are effective in combating microbial infections. The biological potentials of synthesized nanoparticles might be due to the presence of phosphate-solubilizing proteins in extracellular matrix of *F. oxysporum*. To understand the maximum capabilities of synthesized AgNPs from the *F. oxysporum* extracellular matrix, it would be beneficial to analyze the in vivo antimicrobial activities.

## Data Availability

No new data were created or analyzed in this study. Data sharing is not applicable to this article.
